# Opportunistic Assessment of Dental Pathologies in Cervical Computed Tomography Angiography: A Proof of Concept Study

**DOI:** 10.1007/s00062-026-01630-y

**Published:** 2026-02-20

**Authors:** Tim Halstenbach, Maximilian F. Russe, Sabrina Zimmermann, Fabian Cieplik, Rainer Schmelzeisen, Horst Urbach, Wiebke Semper-Hogg, Alexander Rau

**Affiliations:** 1 Department of Operative Dentistry and Periodontology, University Medical Center Freiburg, Freiburg, Germany; 2 Department of Diagnostic and Interventional Radiology, University Medical Center Freiburg, Freiburg, Germany; 3 Department of Oral- and Craniomaxillofacial Surgery, University Medical Center Freiburg, Freiburg, Germany; 4 Department of Neuroradiology, University Medical Center Freiburg, Freiburg, Germany

**Keywords:** Head and Neck, Dental, Stroke, Periodontitis, Angiography

## Abstract

**Objective:**

Computed tomography angiography (CTA) of the head and neck routinely encompasses dentoalveolar structures within the field of view, yet dental findings are rarely reported. Opportunistic detection of dental pathologies could provide valuable information for patient management. This feasability study evaluated the diagnostic potential of CTA for identifying common dental pathologies compared with panoramic radiography (orthopantomogram, OPG).

**Materials and Methods:**

Fifty-seven patients (39% female, mean age: 63 years), who underwent both OPG and head-and-neck CTA within 100 days were retrospectively included. Three experienced dental radiological examiners independently assessed each tooth for periapical radiolucencies, periodontal bone lesions, carious lesions, root residues, retained teeth, and dental implants in cervical CTA and vascular kernel. Cohen’s κ and Fleiss’ κ were calculated to determine intra- and inter-rater-intramodality agreement between OPG and CTA findings.

**Results:**

CTA demonstrated good intermodality agreement with OPG regarding the detection of periapical lesions and periodontal bone defects, root canal-filled, retained teeth, and root residues (κ = 0.60–0.98). Carious lesions demonstrated moderate concordance. OPG identified more periodontal bone defects (mean number of detections per rater: CTA: 141.3; OPG: 173) and carious lesions (CTA: 48; OPG: 77; both *p* < 0.001).

**Conclusions:**

This systematic comparison of CTA and OPG demonstrated the feasability of CTA for opportunistic screening of dental status. While CTA can not replace dedicated dental imaging and clinical assessment, integrating dental evaluation into CTA interpretation may support early detection of oral infection sources and streamline interdisciplinary patient management in neurovascular imaging workflows.

**Supplementary Information:**

The online version of this article (10.1007/s00062-026-01630-y) contains supplementary material, which is available to authorized users.

## Key Points

- *Question:* Does Computed Tomography Angiography (CTA) of the head and neck provide insights into the dento-alveolar pathologies comparable to standard dental imaging with panoramic radiographs?
- *Findings: *Head and neck CTA allowed for comparable identification of periodontal bone defects, periapical lesions, root canal fillings and retained teeth with OPG.
- *Clinical relevance:* Opportunistic dental assessment of head and neck CTA allows for the screening of the dental status and may allow for the identification of dental infectious foci in stroke patients.

## Introduction

Odontogenic diseases are a frequent cause of head and neck infections and can exacerbate systemic infections and inflammatory responses. Apart from clinical inspection, dental diagnostics of odontogenic infection largely relies on radiographic evaluation. Cross-sectional imaging modalities such as computed tomography (CT) and cone-beam computed tomography (CBCT) have increasingly been utilized for dental and maxillofacial diagnostics, providing high spatial resolution and three-dimensional assessment [1–3]. Non-dental specific imaging of the head and neck region routinely encompasses the maxillofacial skeleton and dentition within the field of view. Consequently, such could provide valuable opportunistic information regarding dental health and potential odontogenic sources of infection. Panoramic radiography (orthopantomography, OPG) is widely employed in dentistry as a low-threshold overview examination of dentoalveolar structures, giving the base for further diagnostics [4, 5]. It relies on a rotational, layer-selective projection technique but is limited by two-dimensional projection and inherent geometric distortions [6]. OPG therefore cannot be considered an ideal diagnostic tool for all dental pathologies; however, it is routinely applied as a baseline examination owing to its wide availability, comparatively low radiation exposure, and comprehensive overview of the dental structures.

The most prevalent radiologically detectable dental infection foci are periodontal and periapical lesions, each with distinct pathophysiological and imaging characteristics. Periodontal defects originate from chronic inflammation of the supporting structures of the teeth, including the gingiva, periodontal ligament, and alveolar bone [7]. Periodontal defects are characterized radiologically by horizontal or vertical bone loss, widening of the periodontal ligament space, and denudation of the furcation of multirooted teeth [5–7].

In contrast, endodontic lesions frequently arise from pulp necrosis as a consequence of bacterial invasion. Diffusion of toxins, inflammatory cytokines, and bacteria over the root apex leads to apical periodontitis, granulomas or periapical cysts, all of which are radiologically characterized by the formation of periapical radiolucencies [3]. Carious lesions and retained teeth may serve as additional indicators of oral disease burden.

In the acute workup of patients with craniocervical trauma or suspected stroke, CT angiography (CTA) facilitates the detection of vascular injuries or pathologies associated with ischemic or hemorrhagic stroke [8]. However, dental structures visualized on CTA are often underreported. Previous studies have shown that incidental dental findings on head and neck CT frequently remain undocumented, despite their potential clinical relevance [9, 10]. Insufficient radiological reports may delay the diagnosis and treatment of dental ailments, which can lead to infectious complications in the rehabilitation after strokes and was found to increase the risk of secondary events [11].

This study is intended as a feasibility study to investigate the potential of CTA for the opportunistic assessment of periodontal, periapical bone and carious lesions, as well as root residues or retained teeth, in comparison to OPG as routine basic dental imaging modality.

## Materials and Methods

### Study Population

**Fig. 1:**
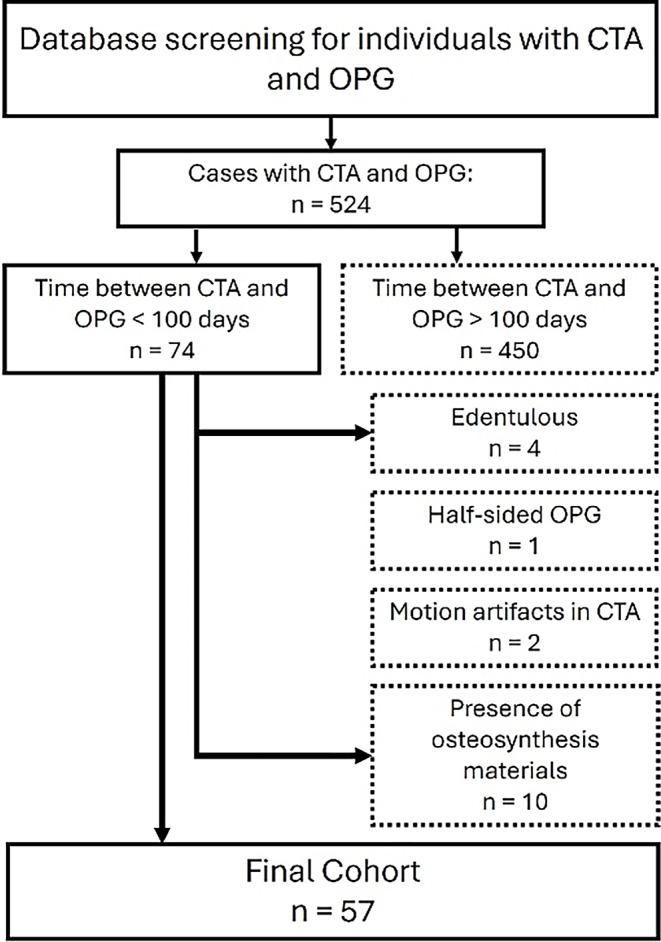
Cohort Flow Chart. Flow chart of patient inclusion and exclusion

The present study was approved by the ethics committee of the University of Freiburg, Germany (Nr. 25-1360-S1-retro) and carried out in accordance with the Declaration of Helsinki and its later amendments. The study was designed as a feasibility study. For radiological dental assessment, OPGs were used as the reference modality. Hence, this retrospective study included adult patients (> 18 years) who underwent both panoramic radiography (OPG) and head-and-neck CT angiography (CTA) with a maximum delay of 100 days within the period of 04/2016 and 01/2025. Patient screening was performed by a neuroradiologist who was not included in the subsequent assessments of dental pathologies (A. R.). Patients had to have a minimum of five remaining teeth or intraosseous dental implants. Cases were dismissed if teeth were extracted, implants were placed, or if other major therapies were performed impacting the dento-alveolar system between CTA and OPG. Cases were also excluded in case of motion artefacts or incomplete depiction of dento-alveolar system in CTA or OPG. Cases with osteosynthesis material projecting over the jaws were excluded, except for dental implants (Fig. 1).

### CTA-Acquisition

CT scans were performed on 64- or 128-detector row CT scanners (Somatom Definition Flash and Definition AS, Siemens Healthineers, Forchheim, Germany). CTA scans were obtained in spiral mode covering the aortic arch to the vertex with the following typical settings: reference tube voltage = 100 kV, reference tube current = 155 mAs, CARE Dose4D, collimation = 128 × 0.6 mm, tube rotation time = 0.28 s, no gantry tilt. Contrast agent was administered via an antecubital vein (16–18 G) with a contrast agent dose = 70 mL, flow rate = 5 mL/s, duration = 14s, followed by a 60 mL saline flush at a flow rate of 5 mL/s.

### OPG-Acquisition

OPGs were obtained using two extraoral imaging systems: VistaPano S/S 2.0 (Dürr Dental, Bietigheim-Bissingen, Germany) and Orthophos XG 3D ready (Dentsply Sirona, Bensheim, Germany). Standard adult panoramic acquisition protocols were applied. For the VistaPano S/S 2.0, exposure parameters were typically within 72–73 kV and 11–12 mAs, with a scan time of approximately 7 s. For the Orthophos XG 3D ready, standard adult settings were typically within 63–69 kV and 8–12 mAs, with a scan time of approximately 14 s. Exposure selection was adapted only to patient body habitus using the preset manufacturer adult programs, with no hardware or software modifications during the study period.

### Image Analysis

Both CTAs and OPGs were examined independently by three dental clinicians with substantial experience in analysis of both OPG and CBCT (with > 15, > 5, and > 4 years of experience in dental imaging analysis, respectively). Raters were blinded to the clinical data of the patients. OPGs and CTAs were evaluated separately in DeepUnity Diagnostic 2.0.2.2 using dedicated workstations with a minimum interval of three weeks between the two sessions. For every tooth of each patient, examiners were asked to record if a periapical, periodontal bone defect, carious lesion or root residue was visible or if the tooth was retained. The presence of dental implants was recorded likewise. For CTA analysis, images were assessed in vascular kernel and 0.75 or 1.0 mm isotropic freely adjustable three-dimensional reformats and hard windowing (center: 400 Hounsfield Units, width: 2000 Hounsfield Units).

**Fig. 2:**
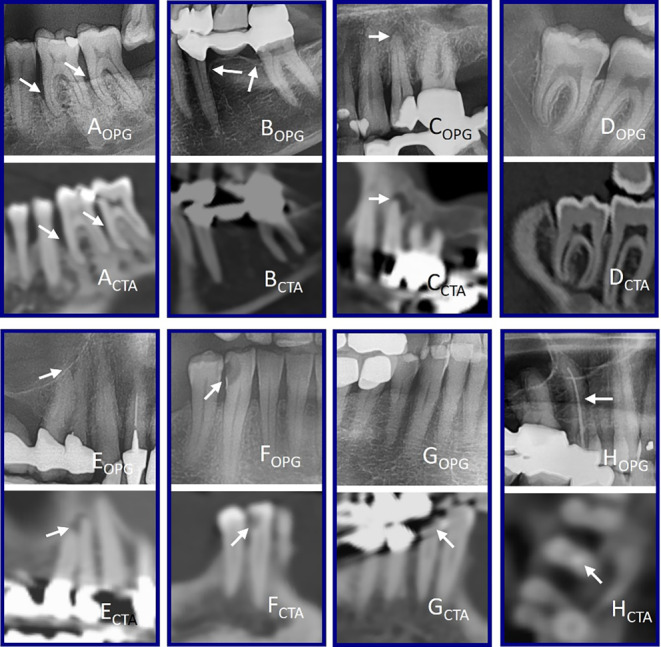
Exemplary dental findings in CTA and OPG. Exemplary dental findings in OPGs (upper image) and the respective CTA (lower image). **a** Arrows mark periodontal intra-bony defects, identified by all raters both in CTA and OPG. **b** OPG reveals periodontal bone defects (identified by all raters) that were not identified in CTA due to beam-hardening artefacts. **c** Arrow marks a periapical radiolucency, that was identified by all raters in both modalities. **d** Third molar identified as partially retained by two raters in OPG and by all raters in CTA. **e** Arrow indicates a periapical radiolucency, identified by 2/3 raters in OPG and by all raters in CTA. **f** Arrows mark carious lesions, which were identified by all raters in both modalities. **g** Arrow marks beam hardening artefacts that were misinterpreted as carious lesions by one rater. **h** Arrow marks RCF identified in OPG by all raters and unidentified by one rater in CTA

Periapical radiolucencies were recorded only when a clearly demarcated radiolucent area exceeding physiological widening of the periodontal ligament space was present, accompanied by loss of lamina dura continuity and/or periapical bone rarefaction. Subtle or equivocal widening of the periodontal ligament space alone was not classified as periapical pathology, particularly in CTA, where spatial resolution and acquisition parameters limit the reliable assessment of minimal changes (Fig. 2). Vertical bone defects and exposure of the furcation were classified as periodontal bone defects, whereas generalized horizontal bone loss was not evaluated due to its limited specificity and potential association with previous disease or lower disease stages [12]. Carious lesions were documented when a distinct coronal radiolucency was present, and teeth were categorized as a root residue if the majority of the crown was destroyed. Both partially and fully impacted teeth were classified as retained.

### Statistical Analysis

Statistical analysis was performed in R (V. 4.5.1; https://www.r-project.org/) and RStudio (V. 2025.05.1). For intra-rater-intermodality comparisons (OPG vs. CTA), Cohens κ was calculated for the individual defect groups (periapical radiolucencies, periodontal defects, root canal-filled teeth, carious lesions, root residues, retained teeth, dental implants). Interpretation of κ statistics in tooth-wise analyses must consider the strong prevalence imbalance inherent to dental datasets, with a large proportion of normal teeth. To address this, we additionally report the prevalene-adjusted bias-adjustes κ (PABAK) and positive agreement.

For the assessment of inter-rater-intramodality agreement in CTA and OPG, Fleiss’ κ and positive agreement for the pairwise comparisons between the raters was calculated. Paired Wilcoxon signed-rank tests with subsequent Benjamini-Hochberg correction for multiple testing was used to assess the prevalence of different findings in OPG and CTA across all raters. Intra-rater-intermodality and inter-rater-intramodality comparisons were performed for different tooth locations: upper and lower jaw, anterior teeth (including canines and incisors), and posterior teeth (including molars and premolars). To assess the intra-rater-intramodality agreement, one rater (rater 1) reassessed CTAs after four months and Cohens κ, PABAK and positive agreement were calculated. Significance threshold was set to *p* ≤ 0.05 and κ was interpreted according to Landis and Koch: κ ≤ 0.2 = poor, 0.21–0.4 = fair, 0.41–0.6 = moderate, 0.61–0.8 = good, 0.81–1 = very good [10].

## Results

A total of 57 cases were enrolled in the study, including 22 female (39%) and 35 (61%) male individuals. A flow chart on cohort assembly is given in Fig. 1. Age at the time of the first imaging ranged from 21 to 94 years, with a mean age of 63 ± 18 years. Mean delay between OPG and CTA was 26 ± 28 days. Of all CTAs, 37 (65%) were performed in the context of stroke diagnostics, 18 (32%) were indicated due to trauma, and 2 (3%) were obtained for oncological evaluation. For every examined case, 32 tooth positions were evaluated, resulting in 1824 examined tooth positions containing a total of 1248 teeth, with an average of 22 teeth per patient (median: 21.9, IQR: 18–27).

### Intra-Rater-Intermodality Analysis

**Fig. 3:**
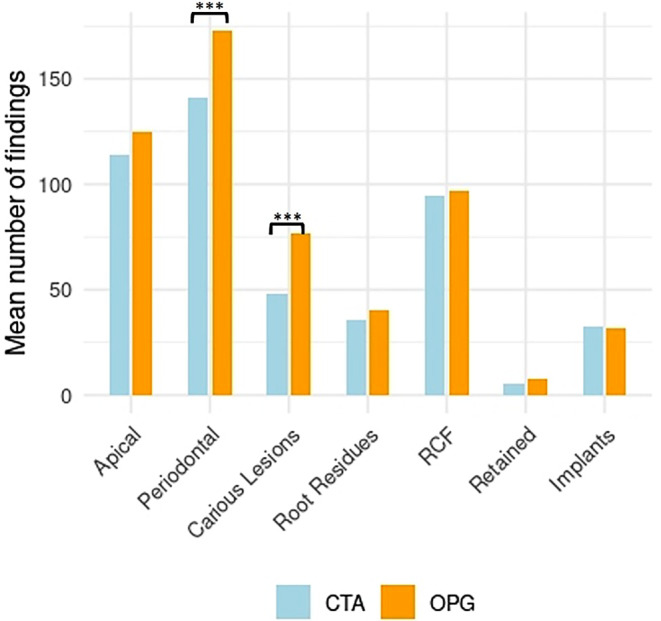
Mean Number of Findings across all Raters. Bar plots for the prevalence of different radiological findings in head and neck angiography (CTA) and Orthopantomogram (OPG). Mean number of detected lesions across all three raters. Wilcoxon signed-rank test indicated significantly higher detection rates for periodontal and carious lesions in OPGs. *Apical* periapical radiolucencies; *Periodontal* periodontal bone defects; *RCF* root canal-filled; *Retained* partially and fully retained teeth; *Implants* dental Implants

Across all raters, no significant differences were observed for the identification of apical lesions (mean number of detections per rater: CTA: 114; OPG: 125), root residues (CTA: 35.3; OPG: 40) or retained teeth (CTA: 5.3; OPG: 7.7) upon comparing CTA with OPG. Assessment of OPGs for periodontal bone and carious lesions lead to significantly more findings than the assessment of CTAs (periodontal: CTA: 141.3; OPG: 173; carious lesions: CTA: 48; OPG: 77; both *p* < 0.001) (Fig. 3).

The results of the intra-rater-intermodality analysis are summarized in Table 1. Periodontal bone lesions were the most common defect types, with 124 to 199 detected defects across all CTAs and OPGs analysized. Intra-rater-intermodality agreement was moderate (rater 3) to good (rater 1 and 2). For periodontal bone defects of teeth of the lower jaw and for anterior teeth, the intra-rater-intermodality agreement was lower than for posterior teeth and ones from the upper jaw (supplementary table 1). Detection of periapical radiolucencies showed good to very good intra-rater-intermodality agreement for all raters and all tooth positions. RCF and dental implants were detected with high intra-rater-intermodality agreement (κ > 0.9) across all raters and tooth locations. Compared to the other observed categories, carious lesions showed the lowest, mostly moderate intra-rater-intermodality agreement. However, root residues were detected with good to very good agreement. Positive agreement was lowest for carious lesions in both modalities (OPG: 0.51–0.79; CTA: 0.57–0.81). For other clinical relevant findings, positive agreement values indicated robust concordance. Exemplary cases of positive and missed identification in both modalities are depicted in Fig. 2 and supplementary figure 1.

**Table 1:** Teeth-wise Intra-rater-intermodality correlation

	Rater 1				Rater 2				Rater 3			
	Positive in OPG/Positive in CTA	PABAK	Positive Agreement	κ (95%-CI)	Positive in OPG/Positive in CTA	PABAK	Positive Agreement	κ (95%-CI)	Positive in OPG/Positive in CTA	PABAK	Positive Agreement	κ (95%-CI)
Periapical Radiolucencies	90/100	0.947	0.747	0.734 (0.661–0.799)	162/134	0.950	0.845	0.831 (0.782–0.879)	123/108	0.920	0.684	0.663 (0.589–0.731)
Periodontal Bone Defects	145/124	0.922	0.736	0.715 (0.651–0.773)	199/159	0.895	0.732	0.703 (0.647–0.758)	175/141	0.875	0.639	0.605 (0.536–0.667)
Carious Lesions	43/30	0.968	0.603	0.595 (0.443–0.712)	110/65	0.933	0.651	0.635 (0.543–0.715)	78/49	0.933	0.520	0.503 (0.385–0.603)
Root Residues	34/34	0.997	0.769	0.85 (0.747–0.935)	44/37	0.988	0.864	0.861 (0.765–0.938)	42/35	0.981	0.779	0.775 (0.651–0.875)
RCF	96/91	0.988	0.941	0.938 (0.898–0.972)	97/97	0.996	0.979	0.978 (0.952–0.995)	97/95	0.987	0.938	0.934 (0.895–0.969)
Retained Teeth	8/5	0.989	0.853	0.768 (0.399–1)	7/5	0.998	0.833	0.833 (0.5–1)	8/6	0.996	0.714	0.713 (0.333–1)
Dental Implants	32/32	0.999	0.985	0.984 (0.947–1)	32/32	0.998	0.969	0.968 (0.919–1)	32/32	0.998	0.969	0.968 (0.919–1)

Three raters examined CTA and OPG of 57 individuals

*κ* Cohens κ; *PABAK* Prevalence-Adjusted Bias-Adjusted Kappa

### Inter-Rater-Intramodality Analysis

Overall, the inter-rater-intramodality agreement was high for both CTAs and OPGs (Table 2). However, with the exception of retained teeth, CTAs demonstrated slightly better agreement. For both RCF and dental implants, Fleiss’ κ indicated near perfect agreement between CTAs and OPGs. Inter-rater-intramodality agreement for the detection of periapical and periodontal lesions was good in both imaging modalities, with slightly lower agreement for mandibular and posterior teeth (supplementary table 2). The lowest, but still good agreement was found for carious lesions (OPG: κ = 0.65; CTA: κ = 0.67).

**Table 2:** Inter-rater-intramodality agreement

	Orthopanthomography				CT Angiography			
		Positive Agreement				Positive Agreement		
	Fleiss κ	1 vs. 2	2 vs. 3	1 vs. 3	Fleiss κ	1 vs. 2	2 vs. 3	1 vs. 3
Periapical Radiolucencies	0.708 (0.652–0.764)	0.651	0.704	0.814	0.785 (0.734–0.835)	0.769	0.779	0.843
Periodontal Bone Defects	0.728 (0.681–0.774)	0.674	0.756	0.824	0.78 (0.734–0.826)	0.763	0.777	0.847
Carious Lesions	0.647 (0.57–0.725)	0.510	0.645	0.798	0.672 (0.578–0.766)	0.568	0.633	0.807
Root Residues	0.864 (0.797–0.93)	0.795	0.868	0.930	0.865 (0.795–0.936)	0.845	0.812	0.944
RCF	0.949 (0.922–0.976)	0.953	0.943	0.959	0.955 (0.93–0.981)	0.936	0.946	0.990
Retained Teeth	0.956 (0.871–1)	0.933	1	0.933	0.812 (0.599–1)	0.800	0.909	0.727
Dental Implants	0.979 (0.949–1)	0.969	0.969	1	0.99 (0.969–1)	0.985	0.985	1

Inter-rater-intramodality agreement for tooth-wise assessments. Overall agreement across all raters was assessed using Fleiss’ κ. Pairwise rater agreement (Rater 1 vs. 2; Rater 2 vs. 3; Rater 1 vs. 3) is reported using positive agreement

### Intra-Rater-Intramodality Analysis

To assess the reproducibility of CTA analysis for dental findings, one reader reassessed all CTA datasets after 4 months. Overall, very good agreement and positive agreement rates of > 0.8 were found for all defect types (Table 3).

**Table 3:** Intra-rater-intramodality comparison

	Kappa	PABAK	Positive Agreement	Negative Agreement
Periapical Radiolucencies	0.847	0.974	0.854	0.993
Periodontal Bone Defects	0.881	0.976	0.888	0.994
Carious Lesions	0.883	0.993	0.885	0.998
Root Residues	0.887	0.993	0.889	0.998
RCF	0.947	0.99	0.949	0.997
Retained Teeth	0.889	0.999	0.889	1
Dental Implants	1	1	1	1

Intra-rater-intramodality correlation of CTA readings performed by rater 2

*Kappa* Cohens Kappa. *PABAK* Prevalence-Adjusted Bias-Adjusted Kappa

## Discussion

This study investigated the feasibility of CTA in comparison to OPG for the first line assessment of dental findings. We found that CTA assessment of periapical and periodontal bone lesions, root canal-filled, root residues and retained teeth showed comparable results to OPG with good to very good inter-rater-intramodality agreement. This corroborates the potential of CTA for opportunistic dental diagnostics.

While previous evidence on the diagnostic potential of CTA for dental pathologies was scarce, we herewith present a systematic comparison of CTA with standard dental imaging. This is of significance, as dental diseases are frequently underreported in head and neck CT examinations [9, 10]. Dental examinations in CTA is especially relevant in the context of neurovascular diseases, as several epidemiological and clinical studies have reported associations between poor oral health and an elevated risk of stroke [13–15]. Both periodontal and endodontic infections have been assumed to exert systemic effects through the persistent release of pro-inflammatory mediators and dissemination of oral pathogens into the bloodstream [16, 17]. The examination of CTAs of patients with neurovascular diseases could not only allow for insights into the individual dental disease load but also provide a valuable source for future research by allowing for longitudinal and population-based analyses of oral health status.

We observed high inter-rater-intramodality and intra-rater-intermodality agreement for the detection of periodontal bone defects between imaging modalities; however, a notable proportion of defects identified on OPG could not be verified on CTA and vice versa. In general, sensitivity for the detection of periodontal bone defects using two-dimensional imaging has been described with high variance, ranging from 30% to 70% [18, 19]. In contrast, three-dimensional imaging, including CBCT, can facilitate precise assessments of periodontal bone defects due to better depiction of the oro-vestibular dimension [1]. In this study, however, inter-rater intramodality variances for the detection of periodontal bone defects in CTA were comparable to OPG, rather than to CBCT. One possible explanation is increased restoration-induced artefacts in CT scans which can hinder detection of periodontal bone defects. This effect may be more pronounced in the mandible, where deviation from the central imaging plane likely increases artefact projection onto the alveolar bone, potentially explaining the slightly lower intra-rater-intermodality agreement for posterior teeth and ones from the lower jaw as observed in this study [20]. Lastly, intra-rater-intramodality agreement was high, too. Consequently, CTA provides initial information on periodontal status but cannot replace intraoral or three-dimensional dental imaging and clinical assessment.

Our study focused on the identification of infrabony periodontal defects and furcation involvement, which are associated with advanced disease stages and progressive inflammatory destruction [12, 21, 22]. Horizontal bone loss patterns, however, are also influenced by past disease or less advanced stages of disease [12]. By restricting the analysis to vertical and furcation defects, this study aimed to capture periodontal changes that are more likely to indicate active or advanced inflammatory processes, rather than overall periodontal disease burden.

Inter-rater-intramodality agreement for the detection of periapical lesions was good to very good for both CTAs and OPG and within the range of previous studies on OPG, however CTA showed a tendency to higher inter-rater agreement [21, 22]. Previous investigations on the detection of periapical radiolucencies with CBCT have demonstrated the advantage of three-dimensional imaging modalities [23]. In our study, this may also be reflected by the high intra-rater-intramodality agreement for all defect types. This contrasts with reports on OPG, where intra-rater-intramodality agreement for periapical radiolucencies has been described as only moderate, reflecting the known interpretative variability of subtle periapical findings on OPG [11]. The higher repeatability observed in our CTA reread likely reflects the restriction to clearly demarcated periapical bone changes and the three-dimensional depiction of the periapical region. A noticeable variance was observed regarding the intra-rater-intermodality agreement of the three different raters for periapical radiolucencies. Similar heterogeneities have been reported before and underline that the detection and classification of dental pathologies are not solely dependent on image quality or modality but also influenced by the subjective interpretation of the examiner [21, 22]. These findings suggest that, for manifest periapical pathology, CTA assessment can be reproducible, even under non-dental acquisition conditions. Nevertheless, high reproducibility should not be equated with diagnostic accuracy, and subtle or early lesions may remain below the reliable detection threshold of CTA. Future research should compare the potential of CTA with CBCT as this constitutes the modality of highest sensitivity.

The lowest, yet still moderate to good agreement was observed for the detection of carious lesions in both modalities. It has been emphasized before that carious detection based on CT and CBCT is hampered by the presence of beam-hardening and scatter artefacts caused by restorative dental materials [24, 25]. While OPGs are less susceptible to artefacts arising from high-density restorative materials, geometric distortion and overlap in the approximal areas may hinder the accurate detection of caries [5, 26].

The following limitations need to be considered. First, no clinical data was included in the analysis. For periodontal diseases, clinical examinations remain central to conclusively determine the extent of the disease [12]. In the present study, mean delay between CTA and OPG was 26 days, and an independent examiner confirmed that no major dental therapies were performed between the two imaging assessments. However, variations in the timing of image acquisition may influence the detectability of certain lesions. In this study, image analysis was performed by experienced dental clinicians to demonstrate feasibility. Although applicability to routine neuroradiology practice remains unassessed, the findings indicate that head and neck CTA depicts a range of dental pathologies, which warrant systematic consideration during image evaluation. Further, CTAs were compared only to OPGs, which, despite their high relevance in dental practice, are limited in their sensitivity for some dental lesions, including periapical and carious lesions [3, 25]. The detection of dental pathologies is inherently limited by the specific technical and imaging characteristics of each modality, with both CTA and OPG showing strengths and weaknesses depending on the type of defect. Since OPG does not represent a diagnostic gold standard for the investigated dental pathologies, diagnostic accuracy measures such as sensitivity or specificity of CTA cannot be derived from this study. Instead, the results illustrate the feasibility of opportunistic dental assessment on CTA and support its role as a modality capable of providing baseline dental information comparable to OPG, rather than exhaustive lesion detection.

Regarding the variance in the detection of the different parameters, it should further be noted that individual teeth may have been inconsistently allocated to specific tooth positions by different raters, which could have introduced assignment errors and partially accounted for the observed deviations between evaluations. The tooth-wise analysis included a high proportion of normal teeth, which may inflate κ values, particularly for infrequent findings. Therefore, κ should be interpreted in the context of lesion prevalence and positive agreement. Lastly, CTA were reviewed without dental-specific reconstructions (e.g. very hard kernels and small field-of-view), which might facilitate higher performance in the assessment of dental pathologies. As head-and-neck CTA positioning is optimized for neurovascular assessment, it often places the jaws peripherally in the gantry which can hamper visualization of subtle periodontal bone changes, representing a limitation of opportunistic CTA data that is less relevant for dedicated dental CT protocols.

In summary, this systematic comparison of CTA and OPG for detecting periodontal and endodontic disease corroborates the potential of CTA for opportunistic screening of dental status and might be appended to stroke workup. Prompt CTA-based evaluation of oral health may support early detection of treatment needs and streamline interdisciplinary workflows, without the need for additional imaging.

## Supplementary Information

ESM1: Supplementary material 1

## Data Availability

No datasets were generated or analysed during the current study.

## Abbreviations

CTA: Computed Tomography Angiography
CBCT: Cone-Beam Computed Tomography
OPG: Orthopantomogram/panoramic radiograph
