# Supplementary material for: Opportunistic Assessment of Dental Pathologies in Cervical Computed Tomography Angiography: A Proof of Concept Study
Source: Clin Neuroradiol. 2026 Feb 20;36(2):701–9. doi: 10.1007/s00062-026-01630-y (PMC13319318; doi:10.1007/s00062-026-01630-y)
Supplement: Supplementary file 1 — ESM1: Supplementary material 1 [file 62_2026_1630_MOESM1_ESM.pdf]

Supplementary Material

Title: Opportunistic Assessment of Dental Pathologies in Cervical Computed Tomography Angiography: A Proof of Concept Study

Authors: Tim Halstenbach; Maximilian F. Russe; Sabrina Zimmermann; Fabian Cieplik; Rainer Schmelzeisen; Horst Urbach; Wiebke Semper-Hogg; Alexander Rau

Journal: Clinical Neuroradiology

Corresponding Author: Tim Halstenbach. Department of Operative Dentistry and Periodontology, Medical Center and Faculty of Medicine, University of Freiburg, Hugstetter Straße 55, 79106 Freiburg, Germany. Tim.halstenbach@uniklinik-freiburg.de

Supplementary Figure 1: Additional exemplary findings in OPG and CTA

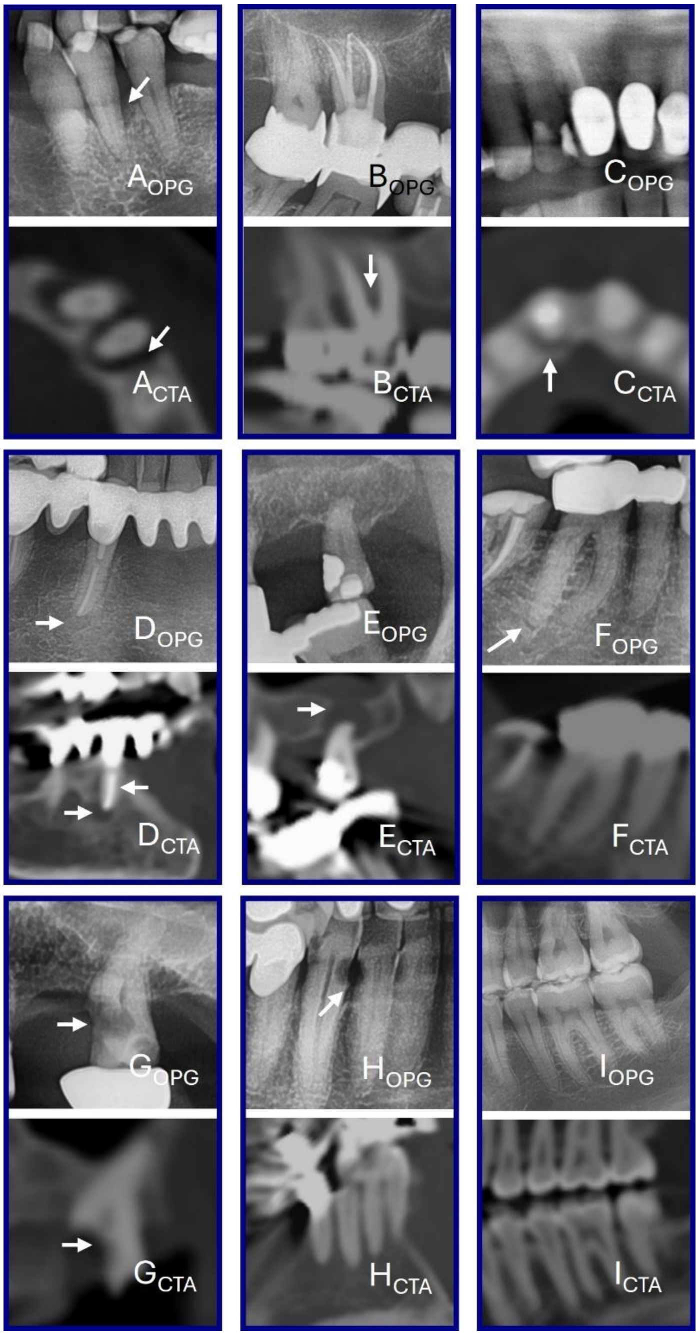

Exemplary dental findings in OPGs (upper image) and the respective CTA (lower image). A: Arrows mark periodontal intra-bony defects, identified by all raters both in CTA and OPG. B: Arrow marks a periodontal bone defect (furcation defect), that was identified by one rater in OPG and by all raters in CTA. C: Arrow marks a periodontal bone defect that was identified by 2/3 raters in CTA and by non in OPG. D: Arrow marks a periapical radiolucency, that was identified by all raters in both modalities. E: Arrow marks a periapical radiolucency, that was not identified by any rater in OPG but by all raters in CTA. F: 2/3 raters marked a periapical radiolucency (arrow) in OPG, which was not corroborated in CTA. G: Arrow marks a carious lesions, which was identified by all raters in both modalities. H: Arrow marks carious lesions that were identified in OPG by all raters but by none in CTA due to beam-hardening artefacts. I: Overlay of posterior teeth due to projection in OPG limits the ability to identify approximal carious lesions.

Supplementary Table 1: Intrarater Correlations across different tooth positions

		**Rater 1**		**Rater 2**		**Rater 3**	
		**Cohens κ**	**CI (95%)**	**Cohens κ**	**CI (95%)**	**Cohens κ**	**CI (95%)**
**Upper Jaw**	Periapical Radiolucencies	0.715	(0.62-0.803)	0.803	(0.736-0.865)	0.647	(0.554-0.735)
	Periodontal Bone Defects	0.712	(0.618-0.796)	0.792	(0.721-0.861)	0.647	(0.559-0.732)
	Carious Lesions	0.563	(0.348-0.749)	0.521	(0.378-0.643)	0.375	(0.22-0.519)
	Root Residues	0.86	(0.722-0.958)	0.794	(0.642-0.909)	0.658	(0.476-0.795)
	Root Canal-Fillings	0.92	(0.855-0.969)	0.972	(0.937-1)	0.933	(0.879-0.977)
	Dental Implants	0.974	(0.907-1)	0.946	(0.86-1)	0.946	(0.86-1)
**Lower Jaw**	Periapical Radiolucencies	0.762	(0.626-0.869)	0.871	(0.798-0.935)	0.686	(0.542-0.786)
	Periodontal Bone Defects	0.719	(0.625-0.805)	0.622	(0.531-0.702)	0.56	(0.45-0.659)
	Carious Lesions	0.624	(0.439-0.784)	0.743	(0.635-0.833)	0.644	(0.491-0.765)
	Root Residues	0.831	(0.613-0.965)	0.967	(0.878-1)	0.965	(0.874-1)
	Root Canal-Fillings	0.961	(0.912-1)	0.987	(0.957-1)	0.935	(0.867-0.985)
	Dental Implants	1	(1-1)	1	(1-1)	1	(1-1)
**Anterior Teeth (Incisors and Canines)**	Periapical Radiolucencies	0.83	(0.712-0.923)	0.802	(0.7-0.888)	0.65	(0.503-0.782)
	Periodontal Bone Defects	0.718	(0.602-0.814)	0.647	(0.545-0.74)	0.592	(0.467-0.693)
	Carious Lesions	0.703	(0.49-0.88)	0.658	(0.51-0.777)	0.588	(0.415-0.739)
	Root Residues	0.887	(0.709-1)	0.864	(0.708-0.972)	0.686	(0.437-0.867)
	Root Canal-Fillings	0.936	(0.861-0.987)	0.985	(0.949-1)	0.952	(0.886-1)
	Dental Implants	1	(1-1)	1	(1-1)	1	(1-1)

**Posterior Teeth (Premolars and Molars**	Periapical Radiolucencies	0.693	(0.596-0.775)	0.843	(0.785-0.895)	0.667	(0.579-0.756)
	Periodontal Bone Defects	0.713	(0.635-0.79)	0.732	(0.66-0.795)	0.612	(0.529-0.688)
	Carious Lesions	0.516	(0.305-0.683)	0.618	(0.498-0.723)	0.443	(0.296-0.583)
	Root Residues	0.826	(0.683-0.937)	0.86	(0.739-0.956)	0.82	(0.681-0.932)
	Root Canal-Fillings	0.939	(0.89-0.981)	0.975	(0.944-1)	0.925	(0.872-0.966)
	Dental Implants	0.98	(0.929-1)	0.959	(0.891-1)	0.959	(0.891-1)

Supplementary Table 2: Interrater correlation for different tooth locations.

		Fleiss κ	95%-CI	Fleiss κ	95%-CI
**Upper Jaw**	Periapical Radiolucencies	0.717	(0.648-0.785)	0.807	(0.745-0.868)
	Periodontal Bone Defects	0.784	(0.724-0.843)	0.782	(0.719-0.845)
	Carious Lesions	0.665	(0.562-0.768)	0.648	(0.498-0.798)
	Root Residues	0.857	(0.772-0.941)	0.805	(0.695-0.915)
	Root Canal-Fillings	0.943	(0.906-0.98)	0.954	(0.92-0.988)
	Dental Implants	0.964	(0.915-1)	0.982	(0.948-1)
**Lower Jaw**	Periapical Radiolucencies	0.687	(0.59-0.785)	0.751	(0.665-0.836)
	Periodontal Bone Defects	0.675	(0.605-0.746)	0.778	(0.71-0.845)
	Carious Lesions	0.627	(0.51-0.745)	0.689	(0.568-0.81)
	Root Residues	0.876	(0.768-0.985)	0.953	(0.887-1)
	Root Canal-Fillings	0.957	(0.919-0.995)	0.957	(0.92-0.995)
	Dental Implants	1	(1-1)	1	(1-1)
**Anterior Teeth** (Incisors and Canines)	Periapical Radiolucencies	0.712	(0.61-0.814)	0.805	(0.716-0.895)
	Periodontal Bone Defects	0.651	(0.562-0.74)	0.794	(0.715-0.872)
	Carious Lesions	0.609	(0.479-0.739)	0.691	(0.557-0.826)
	Root Residues	0.933	(0.858-1)	0.807	(0.665-0.95)
	Root Canal-Fillings	0.969	(0.934-1)	0.979	(0.949-1)
	Dental Implants	1	(1-1)	1	(1-1)
**Posterior Teeth** (Premolars and Molars)	Periapical Radiolucencies	0.705	(0.638-0.773)	0.776	(0.715-0.837)
	Periodontal Bone Defects	0.768	(0.715-0.821)	0.773	(0.717-0.83)
	Carious Lesions	0.672	(0.576-0.768)	0.655	(0.522-0.787)
	Root Residues	0.82	(0.723-0.918)	0.896	(0.82-0.973)
	Root Canal-Fillings	0.938	(0.902-0.975)	0.943	(0.908-0.978)
	Dental Implants	0.973	(0.935-1)	0.987	(0.96-1)
